# Geographical Distribution and Molecular Characterization of *Dirofilaria* spp. in Dogs Across Kazakhstan

**DOI:** 10.3390/ani16162612

**Published:** 2026-08-20

**Authors:** Tanzilya Almysheva, Rabiga Uakhit, Ainura Smagulova, Zhibek Sembaeva, Lyudmila Lider, Bekzhassar Sidikhov, Carlos Hermosilla, Marat Dusmagambetov, Vladimir Kiyan

**Affiliations:** 1 Department of Microbiology and Virology, Astana Medical University, 010000 Astana, Kazakhstan; almysheva.tanzilya@gmail.com (T.A.); dusmagambetov.m@amu.kz (M.D.); 2 Laboratory of Biodiversity and Genetic Resources, National Center for Biotechnology, 010000 Astana, Kazakhstan; erken.uakhitrabiga@gmail.com (R.U.); smagulova0114@gmail.com (A.S.); l.lider@kazatu.kz (L.L.); 3 Department of General Biology and Genomics, L.N. Gumilyov Eurasian National University, 010000 Astana, Kazakhstan; zhibek.sembaeva@yandex.ru; 4 Department of Veterinary Medicine, Saken Seifullin Kazakh Agrotechnical University, 010000 Astana, Kazakhstan; 5 Zhardem-Vet Educational, Scientific, and Production Center, Institute of Veterinary Science and Agrotechnology, Zhangir Khan West Kazakhstan Agrarian and Technical University, 090009 Oral, Kazakhstan; sidihovbm@mail.ru; 6 Institute of Parasitology, Justus Liebig University Giessen, 35392 Giessen, Germany; carlos.r.hermosilla@vetmed.uni-giessen.de; 7 Scientific Center for Biological Research, 010000 Astana, Kazakhstan

**Keywords:** *Dirofilaria repens*, *Dirofilaria immitis*, canine dirofilariosis, molecular epidemiology, phylogenetics, Kazakhstan

## Abstract

Canine dirofilariosis caused by *Dirofilaria immitis* and *Dirofilaria repens* is a vector-borne zoonotic infection of considerable veterinary and public health importance. Despite reports of the circulation of *Dirofilaria* spp. in neighboring countries, data on the molecular prevalence, species composition, and genetic diversity of these parasites in dogs from Kazakhstan remain limited. In the present study, owned, shelter, and stray dogs, which serve as the principal reservoirs of infection, were examined for the presence of microfilariae in blood, and the circulation of *Dirofilaria* spp. was molecularly confirmed. The study was conducted between September 2024 and January 2026 and included 751 canine blood samples collected from 10 regions of Kazakhstan. Initial screening was performed using the modified Knott test. Molecular identification was carried out through amplification of the mitochondrial *cox1* gene, followed by DNA sequencing and comparative analysis with reference sequences available in GenBank. Phylogenetic analysis was performed to assess the genetic relationships of the detected isolates. The overall molecular prevalence of *Dirofilaria* spp. was 3.6% (27/751). The highest infection rate was recorded in the Aktobe region (22.3%), while positive cases were also detected in the Akmola (2.7%), Kostanay (1.7%), and West Kazakhstan (1.8%) regions. Species identification revealed the predominance of *D. repens*, whereas *D. immitis* was detected only in Aktobe region. Phylogenetic analysis confirmed the species identity of all obtained sequences and demonstrated a high degree of genetic similarity with *Dirofilaria* isolates previously reported from Europe and Asia. These findings confirm the circulation of *Dirofilaria* spp. among dogs in several regions of Kazakhstan and indicate the ongoing expansion of canine dirofilariosis in Central Asia. This study provides the first molecular genetic data on *Dirofilaria* spp. in dogs from Kazakhstan and highlights the need for continued epidemiological surveillance and assessment of potential risks to animal and human health.

## Simple Summary

Dogs serve as the primary reservoir hosts for *Dirofilaria* spp., mosquito-borne parasitic nematodes of veterinary and public health importance. Although dirofilariosis is a recognized zoonotic disease, information on its prevalence and distribution among dogs in Kazakhstan remains limited. In this study, dogs from multiple regions of Kazakhstan were examined using molecular methods, including PCR and DNA sequencing. The results confirmed the presence of both *Dirofilaria repens* and *Dirofilaria immitis*, with *D. repens* identified as the predominant species. These findings provide new insights into the geographical distribution and genetic diversity of *Dirofilaria* spp. in Kazakhstan and contribute to veterinary surveillance, assessment of the risk of human infection, and the development of effective dirofilariosis prevention and control strategies.

## 1. Introduction

Dirofilariosis is a zoonotic parasitic disease caused by filarial nematodes of the genus *Dirofilaria* belonging to the family Onchocercidae [1]. Two principal species are distinguished, *Dirofilaria immitis* and *Dirofilaria repens*, as they are the most prevalent and clinically significant species from both pathogenetic and epidemiological perspectives [2]. *D. immitis* causes heartworm disease, the clinical manifestations of which include cough, dyspnea, and signs of heart failure [3].

*Dirofilaria repens*, in contrast, is a subcutaneous parasite that forms subcutaneous nodules and can affect ocular tissues, particularly the conjunctiva [4]. Definitive hosts include domestic and stray dogs and cats, as well as wild carnivores such as foxes, wolves, and lynxes; marine mammals and rodents have also been reported as hosts [5,6]. Both *Dirofilaria* species possess zoonotic potential and can infect humans [7]. *D. repens* is the primary cause of subcutaneous and ocular dirofilariosis in humans throughout Europe and Asia, whereas *D. immitis* may cause pulmonary nodules that are often misdiagnosed as neoplastic lesions [8,9,10]. In recent decades, the incidence of human dirofilariosis has increased in many countries, underscoring the importance of monitoring this parasitic infection from both veterinary and public health perspectives [1,11].

The infection is transmitted by hematophagous dipteran insects, primarily mosquitoes of the genera *Culex*, *Aedes*, and *Anopheles* [12]. During blood feeding, mosquitoes ingest microfilariae, which subsequently develop within the vector from the first-stage larva (L1) to the infective third-stage larva (L3). These infective larvae are then transmitted to a new host during a subsequent blood meal [2,13]. Some studies have suggested that blackflies (family *Simuliidae*) may also participate in the transmission of microfilariae; however, their epidemiological role is considered secondary compared with that of mosquitoes [14,15].

Environmental factors such as stagnant water bodies, rivers, wetlands, and the seasonal activity of vectors play a crucial role in the transmission and maintenance of the infection. The establishment of stable transmission foci is particularly characteristic of regions with warm-temperate climates and well-developed hydrological systems [16].

Furthermore, climate change, accompanied by rising average annual temperatures and the prolongation of the warm season, is contributing to the expansion of mosquito vector ranges and the accelerated development of *Dirofilaria* spp. larvae within their insect hosts [17,18]. As a result, dirofilariosis is increasingly being reported in previously non-endemic regions, including temperate areas where the disease was historically absent [19].

In recent years, canine dirofilariosis has become increasingly widespread in many countries across Europe, Asia, and the Americas, although its prevalence varies considerably depending on geographical and climatic conditions [20,21]. According to studies published between 2023 and 2025, *D. immitis* predominates over *D. repens* in many endemic regions of Europe. For example, the prevalence of *D. immitis* reached 28.6% in Greece (2024), 15.7% in northern Italy (January 2024–January 2025), and 17% in Hungary (March 2023–February 2024) [22,23,24,25]. In the Americas, *D. immitis* infection was reported in 7.4% of dogs in Brazil (February–March 2024) and 6.3% of dogs in the United States (2016–2022), with substantially higher prevalence reported in some southern states [26,27,28,29]. Similarly, high infection rates have been documented in Asia, including China (13.8%, meta-analysis published in 2023), India (22%, 2021–2023), and Thailand (51.5%, 2020–2021) [30,31,32,33,34]. These findings demonstrate that the epidemiology of canine dirofilariasis differs markedly among endemic regions and is strongly influenced by local environmental conditions.

Of particular relevance to the present study is the circulation of *Dirofilaria* spp. in countries neighboring Kazakhstan and throughout Central Asia. In the Russian Federation, molecular studies conducted between 2018 and 2023 reported *D. repens* prevalence of 7.52% in southern and southeastern regions and 7.69% in central and northwestern regions [35,36]. In Uzbekistan (2021–2025), both human dirofilariosis cases, including recurrent *D. repens* infections, and infections in rural and urban dog populations have been reported, indicating an active zoonotic focus [37,38,39,40]. In Kyrgyzstan (2017–2019), molecular studies confirmed the circulation of both *D. immitis* and *D. repens* in dogs [41]. Furthermore, studies from Karakalpakstan (2020–2023) identified *Dirofilaria* larvae in insect vectors, supporting the existence of favorable ecological conditions for parasite transmission in Central Asia [42]. Collectively, these findings suggest that *Dirofilaria* spp. are established throughout the region and highlight the importance of investigating their distribution and genetic diversity in Kazakhstan, where epidemiological data remain limited.

Given the similarity of natural and climatic conditions across the Central Asian states, the presence of transboundary populations of dogs and wild carnivores, and the widespread distribution of potential vectors, there is a high probability that *Dirofilaria* spp. are more widely distributed in Kazakhstan than currently documented in the available literature [22,43].

In Kazakhstan, data on the distribution of dirofilariosis remain limited and are largely restricted to isolated clinical and molecular reports. The first molecular evidence of *D. repens* circulation was obtained from the examination of the heart of a wild wolf, in which adult nematodes were detected within the cardiac chambers [44,45]. Subsequent epizootiological studies further confirmed the circulation of the parasite among stray dogs in the West Kazakhstan Region, where the prevalence of *D. repens* reached 29.4% [46].

Traditionally, microscopic methods for detecting microfilariae, including blood smear examination and the modified Knott test [47,48], have been used for the diagnosis of dirofilariosis. These methods are widely applied in parasitology for primary screening because of their availability and ease of use. However, their diagnostic sensitivity may be limited, particularly in cases of low parasite burden, occult infection, or the absence of circulating microfilariae [49,50]. Therefore, molecular and serological diagnostic approaches, including polymerase chain reaction (PCR) and serological assays, are increasingly employed, as they are considered more sensitive and specific tools for the detection and identification of *Dirofilaria* spp. [51,52,53]. However, comprehensive molecular genetics investigations aimed at assessing the prevalence and genetic diversity of *Dirofilaria* spp. in Kazakhstan have not yet been conducted.

To our knowledge, no large-scale studies encompassing multiple regions of Kazakhstan and simultaneously assessing the prevalence, species composition, and phylogenetic characteristics of *Dirofilaria* spp. in dogs have been reported to date. The absence of such data significantly limits understanding of the current epidemiological situation and the potential risks to both veterinary and public health.

Therefore, the aim of this study is to investigate the molecular prevalence, geographic distribution, and genetic characteristics of *Dirofilaria* spp. in dogs from various regions of Kazakhstan using both microscopic and molecular genetics approaches. The findings will contribute to a better understanding of the epidemiological situation and support the development of effective surveillance and control strategies for this zoonotic infection.

## 2. Materials and Methods

### 2.1. Study Area and Sample Collection

**Figure 1:**
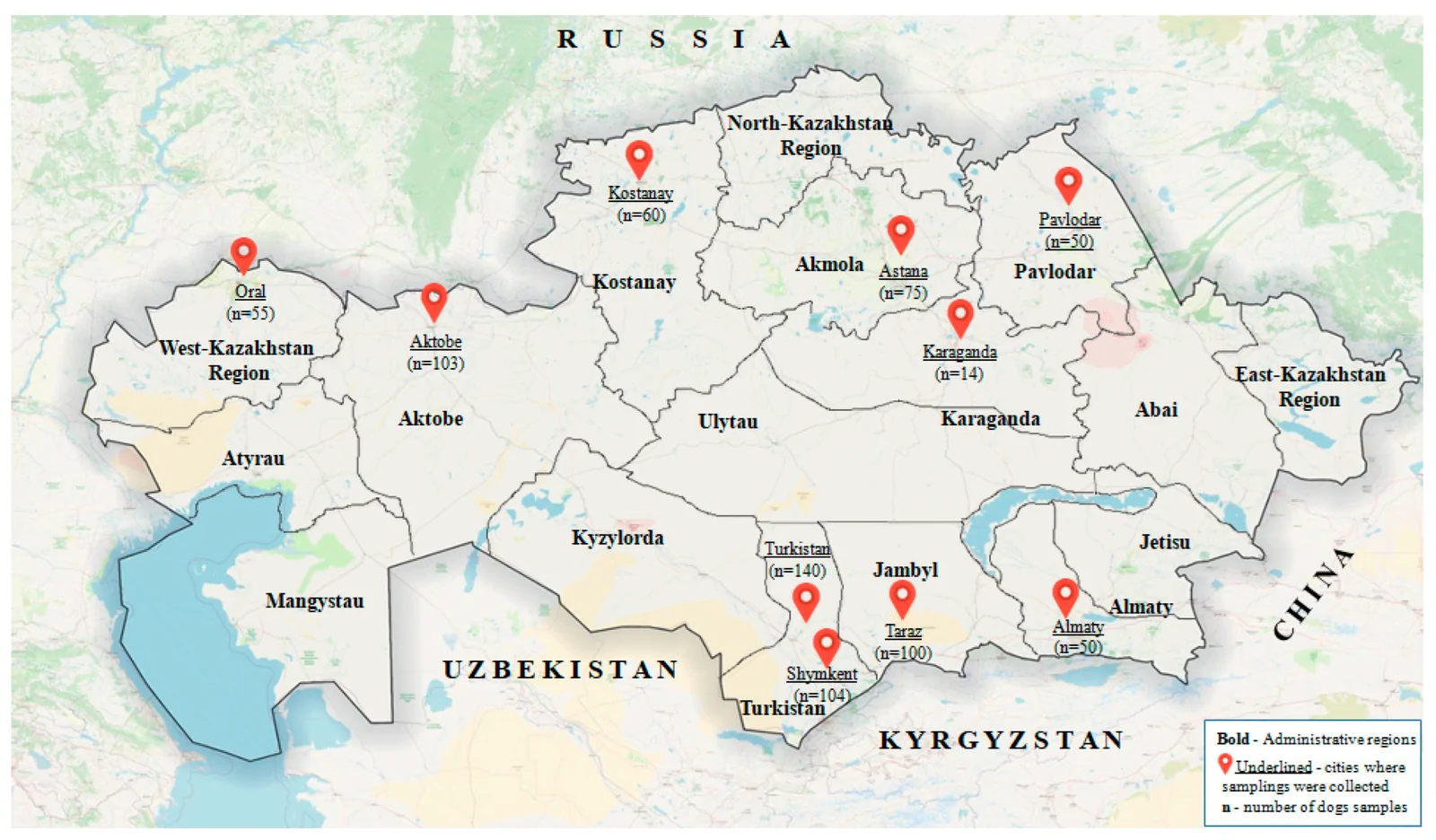
Geographic locations of the sampling sites in Kazakhstan where dog blood samples were collected for the assessment of *Dirofilaria* spp. prevalence.

The study was designed to assess the prevalence and distribution of dirofilariosis in Kazakhstan. Sampling was conducted between September 2024 and January 2026 in ten major urban centers across Kazakhstan: Astana, Almaty, Taraz, Shymkent, Turkestan, Karaganda, Pavlodar, Kostanay, Oral, and Aktobe (Figure 1). The cities were selected to ensure broad geographical coverage of Kazakhstan, taking into account their proximity to major rivers and water bodies that provide favorable environmental conditions for the development of *Dirofilaria* spp. vectors, as well as the accessibility of veterinary clinics, the availability of shelters for stray dogs, and sufficient numbers of animals for the study. All collected samples were transported to the Biodiversity and Genetic Resources Laboratory of the National Center for Biotechnology, Astana, Kazakhstan.

A total of 751 venous blood samples were collected from dogs, with an approximately equal sex distribution (396 males, 325 females, and 30 animals with missing sex data), ranging in age from 6 months to 12 years. Dogs of all age groups, including both owned and stray animals, were included in the study. Of the 751 dogs included in the study, 545 were owned dogs and 206 were stray/shelter dogs. Sampling was convenience-based (opportunistic) and animals were enrolled when they became available through collaborating veterinary clinics or municipal shelters during the study period. Domestic dogs were recruited voluntarily through collaboration with veterinary clinics in the participating cities and by direct contact with dog owners during routine veterinary examinations. Participation was based on the owners’ willingness to provide written informed consent, and no financial incentives were offered. Stray dogs were sampled in collaboration with municipal animal shelters and temporary holding facilities in the participating cities. Animals were enrolled opportunistically during routine veterinary examinations or handling procedures performed by shelter veterinarians. At least one municipal shelter or temporary holding facility participated in each city where stray dogs were sampled. Inclusion criteria comprised dogs of any sex, breed, or age whose owners provided written informed consent (for owned dogs) or that were available for examination in participating municipal shelters (for stray dogs). Dogs were excluded if an adequate and quality blood sample could not be obtained, or if they had received heartworm preventive treatment prior to sampling.

A 2-mL venous blood sample was collected from each dog into EDTA anticoagulant tubes (Zhejiang Gongdong Medical Technology Co. Ltd., Huangyan, Taizhou, China), labeled with a unique identification number, and stored at 4 °C until transportation to the laboratory. Blood sampling was performed following the acquisition of written informed consent from animal owners and shelter or kennel management, where applicable. To obtain information on the individual characteristics and living conditions of the animals, owners and shelter personnel completed standardized questionnaires that included data on the date and location of sampling, age, breed, sex, and body weight, ownership status (owned or stray), and housing conditions of each dog.

### 2.2. Microscopic Examination

For the initial detection of circulating microfilariae in blood samples, the modified Knott test was used. One milliliter (1 mL) of whole blood was mixed with 2% formalin and subsequently centrifuged at 1500× *g* rpm for 5 min. The resulting sediment was transferred onto a glass slide and mixed with methylene blue at a ratio of 1:1 [54,55]. Light microscopic examination (Axio Scope A1, Carl Zeiss Microscopy GmbH, Zeiss, Jena, Germany) was performed at 100× magnification, followed by the evaluation of the morphological characteristics of the microfilariae, including body shape, size, and the morphology of the anterior and posterior ends. The obtained results were used as a primary screening tool prior to molecular analyses.

### 2.3. DNA Extraction Method

Genomic DNA was extracted from whole blood samples using the GeneJET Whole Blood Genomic DNA Purification Kit (Thermo Fisher Scientific, Waltham, MA, USA) according to the manufacturer’s instructions. Briefly, an aliquot of venous blood was subjected to DNA extraction by sequential addition of cell lysis buffer and proteinase K. Following incubation, the lysates were transferred to spin columns and subjected to washing and purification steps. Purified DNA was eluted in 50 μL of 1× TE buffer and stored at −70 °C until further analysis. DNA concentration and purity were assessed using a NanoDrop™ One spectrophotometer (Thermo Fisher Scientific, Waltham, MA, USA).

### 2.4. PCR and Sequencing

For the molecular identification of *Dirofilaria* spp., PCR was performed using primers targeting a fragment of the mitochondrial cytochrome c oxidase subunit I (*cox1*) gene. A fragment of approximately 650 bp was amplified using the primers COI-intF (5′-TGATTGGTGGTTTTGGTAA-3′) and COI-intR (5′-ATAAGTACGAGTATCAATATC-3′) [56,57]. PCR amplification was carried out in a total reaction volume of 25 μL containing 12.5 μL of 2× DreamTaq PCR Master Mix (Thermo Fisher Scientific, Carlsbad, CA, USA), 10 pmol of each primer, nuclease-free water, and 20 ng of template genomic DNA. PCR amplification was performed under the following thermal cycling conditions: 94 °C for 3 min for initial denaturation, and denaturation at 94 °C (30 s), annealing at 52.5 °C (45 s), and extension at 72 °C (1 min) in 30 cycles, with a final extension at 72 °C (7 min). Amplification products were separated by horizontal electrophoresis in a 1.5% agarose gel prepared in 1× TBE buffer. DNA fragments were visualized under UV illumination following staining with ethidium bromide (8 ng/μL).

Samples yielding amplicons of the expected size were considered positive. All PCR-positive products were purified using the Quick PCR Purification Kit (QIAGEN, Germantown, MD, USA) according to the manufacturer’s instructions, and then subjected to sequencing. Sequencing was performed using the BigDye Terminator v3.1 Cycle Sequencing Kit (Applied Biosystems, Foster City, CA, USA) according to the manufacturer’s instructions on a 3730xl DNA Analyzer 96-Capillary Array (Thermo Fisher Scientific, Applied Biosystems, Foster City, CA, USA). The nucleotide sequences obtained in this study were deposited in the GenBank database under accession numbers PZ437830–PZ437856. Species identification was performed by comparison with reference sequences available in GenBank using the BLASTn algorithm (https://www.ncbi.nlm.nih.gov/) (accessed on 13 July 2026).

### 2.5. Estimates of Evolutionary Divergence Between Sequences

Pairwise evolutionary divergence was estimated among the mitochondrial cox1 sequences of *Dirofilaria* species. The dataset comprised 37 nucleotide sequences, including 29 sequences of *D*. *repens*, four sequences of *D. immitis*, two sequences of *D. striata*, and two sequences of *D. ursi*. The *D. repens* dataset included 26 sequences generated in the present study and three reference sequences retrieved from GenBank. One *D. immitis* sequence obtained in this study was analysed together with three reference sequences. The sequences were aligned before analysis, and pairwise evolutionary distances were calculated in MEGA version 11. Distances were expressed as the number of nucleotide substitutions per site.

### 2.6. Phylogenetic Analysis

Multiple sequence alignment was performed using CLUSTALW version 2.1. Phylogenetic analysis was carried out in MEGA version 11 software using the Maximum Likelihood method based on the mitochondrial *cox1* gene sequences [58]. The evolutionary history was inferred by using the Maximum Likelihood method and Tamura-Nei model. The tree with the highest log likelihood (−1645.21) is shown. Initial tree(s) for the heuristic search were obtained automatically by applying Neighbor-Join and BioNJ algorithms to a matrix of pairwise distances estimated using the Tamura-Nei model, and then selecting the topology with superior log likelihood value. A discrete Gamma distribution was used to model evolutionary rate differences among sites (5 categories (+G, parameter = 0.1343)). The reliability of the tree topology was assessed by 100 replicates bootstrap analysis. The final tree was visualised and interpreted to determine the phylogenetic position of the *Dirofilaria* isolates obtained in this study relative to reference sequences available in GenBank. For comparative analysis, reference sequences of *D. repens*, *D. immitis*, *D. ursi*, and *D. striata* were retrieved from GenBank. The sequence of *Onchocerca lupi* was included as an outgroup to root the phylogenetic tree.

### 2.7. Statistical Analysis

Statistical analyses were performed using R software, version R-4.6.1 for Windows (R Foundation for Statistical Computing, Vienna, Austria) [59], with functions implemented in the base stats package. Prevalence was calculated as the number of positive samples divided by the total number examined, and exact 95% binomial confidence intervals were estimated using the Clopper–Pearson method [60]. Associations between *Dirofilaria* spp. positivity and categorical variables were assessed using contingency tables. Before selecting the statistical test, expected cell frequencies were calculated and examined. Pearson’s chi-square test with Yates’ continuity correction [61] was applied to the 2 × 2 tables for sex and living condition because all expected cell frequencies were ≥5. The Fisher–Freeman–Halton exact test [62] was used for city, season, and age class because these tables contained one or more expected cell frequencies below 5. Records with missing sex or age information were excluded from the corresponding analyses. All tests were two-sided, and differences were considered statistically significant at *p* < 0.05.

## 3. Results

### 3.1. Modified Knott’s Method Results

**Figure 2:**
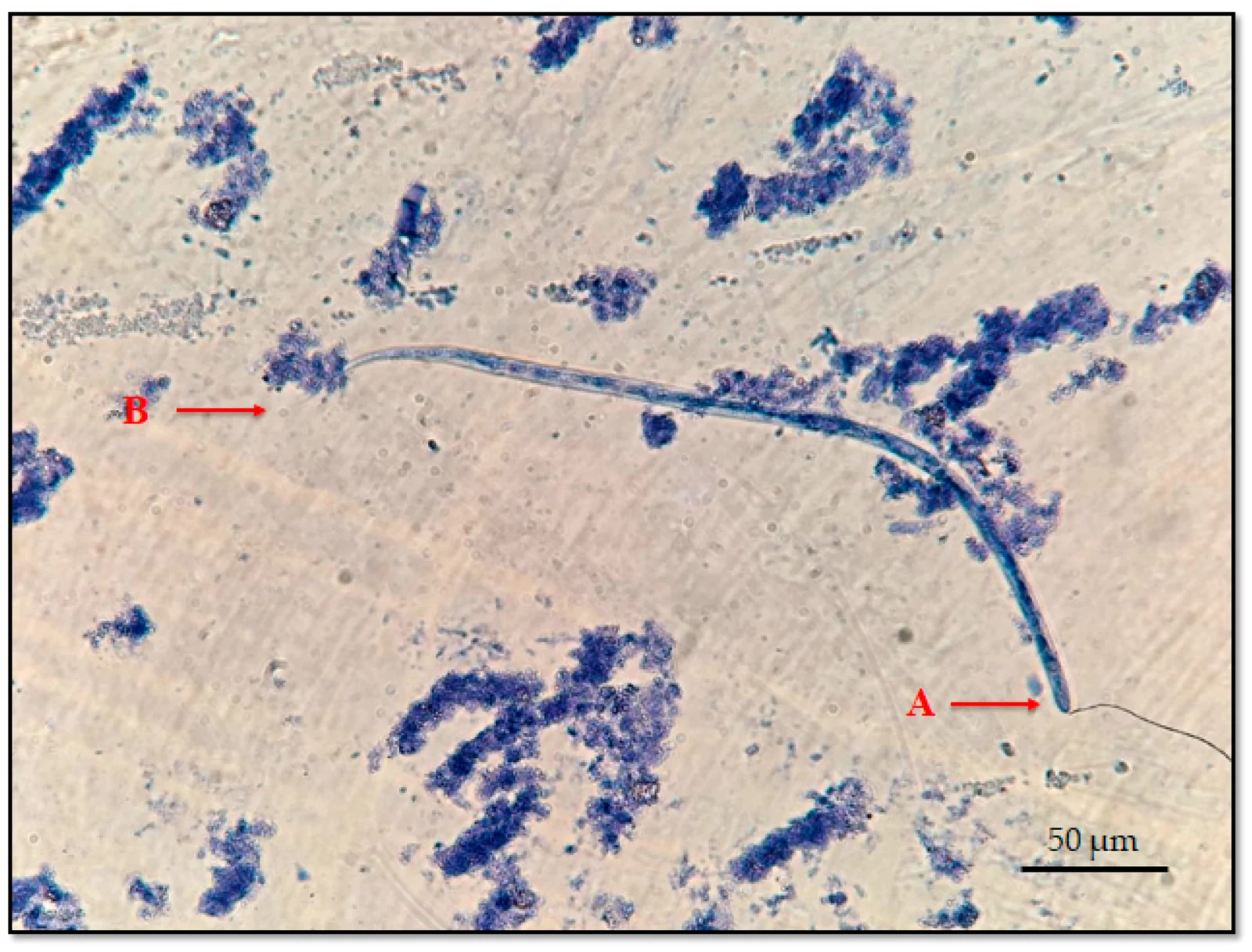
*Dirofilaria* spp. microfilariae detected in dog blood samples: (A) cephalic end; (B) caudal end.

Microscopic examination using the modified Knott method revealed microfilariae in 15 out of 751 examined dogs, corresponding to an overall prevalence of 1.9%. It should be noted that this method was used exclusively for the detection of microfilariae and did not permit species-level identification of *Dirofilaria* spp. (Figure 2).

All positive cases were recorded among dogs sampled in the cities of Astana, Kostanay, Aktobe, and Oral. The highest prevalence was observed in Aktobe, where microfilariae were detected in 12 of 103 dogs (11.7%). In contrast, only one positive sample was identified in each of the other three cities: Astana (1/75; 1.3%), Kostanay (1/60; 1.7%), and Oral (1/55; 1.8%).

### 3.2. Molecular Genetics Identification

**Figure 3:**
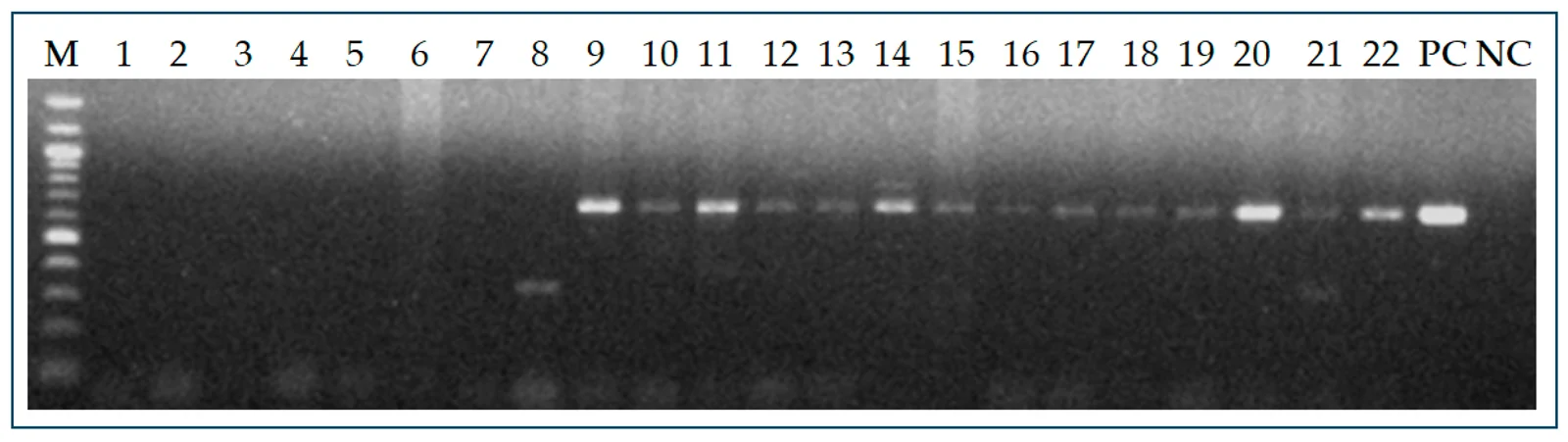
Representative agarose gel electrophoresis of PCR amplification products of the *cox1* gene of *Dirofilaria* spp.: M—DNA ladder (100 bp); lanes 1–22—representative samples; PC—positive control; NC—negative control.

Based on the results of molecular analysis, *Dirofilaria* spp. DNA was detected in 27 of 751 examined dogs, yielding an overall prevalence of 3.6% (95% CI: 2.4–5.2). Species-specific identification revealed *D. immitis* infection in one dog (0.13%) and *D. repens* infection in 26 dogs (3.46%) (Table 1). A representative agarose gel electrophoresis image of the PCR amplification products, including positive and negative controls, is presented in Figure 3.

*Dirofilaria* spp. were detected in 27 of the 751 examined dogs, resulting in an overall prevalence of 3.6% (95% CI: 2.4–5.2%). The frequency of positive results differed significantly among the investigated cities (Fisher–Freeman–Halton exact test, *p* < 0.0001). The highest prevalence was recorded in Aktobe, where 23 of 103 dogs were positive (22.3%; 95% CI: 14.7–31.6%). Considerably lower prevalence was observed in Astana (2.7%), Oral (1.8%), and Kostanay (1.7%), while no positive samples were detected in Almaty, Shymkent, Turkestan, Taraz, Karaganda, or Pavlodar.

A significant association was also identified between *Dirofilaria* spp. positivity and season (Fisher–Freeman–Halton exact test, *p* < 0.0001). Most positive samples were collected in winter, when 25 of 297 dogs were positive, corresponding to a prevalence of 8.4% (95% CI: 5.5–12.2%). In contrast, prevalence was 0.6% in both spring and summer, and no positive samples were recorded in autumn.

Living conditions were strongly associated with infection status (χ^2^ = 43.97, df = 1, *p* < 0.0001). Prevalence among shelter dogs reached 11.2% (23/206; 95% CI: 7.2–16.3%), compared with only 0.7% among companion dogs (4/545; 95% CI: 0.2–1.8%). Significant differences were also observed among age classes (Fisher–Freeman–Halton exact test, *p* < 0.0001). No positive cases were detected among dogs aged 6–12 months, whereas prevalence was 1.2% in dogs aged 12 months to three years and 7.9% in dogs older than three years. By contrast, positivity did not differ between males and females (3.8% and 3.7%, respectively; χ^2^ = 0.00, df = 1, *p* = 1.000).

All samples positive by the modified Knott method were also positive by PCR, while PCR additionally identified 12 positive samples, demonstrating its higher analytical sensitivity (Table 2). These findings highlight the higher diagnostic sensitivity of PCR-based methods compared with conventional microscopic examination and demonstrate the importance of molecular diagnostics for epidemiological studies of canine dirofilariosis.

### 3.3. Phylogenetic Relationships of Dirofilaria Isolates

**Figure 4:**
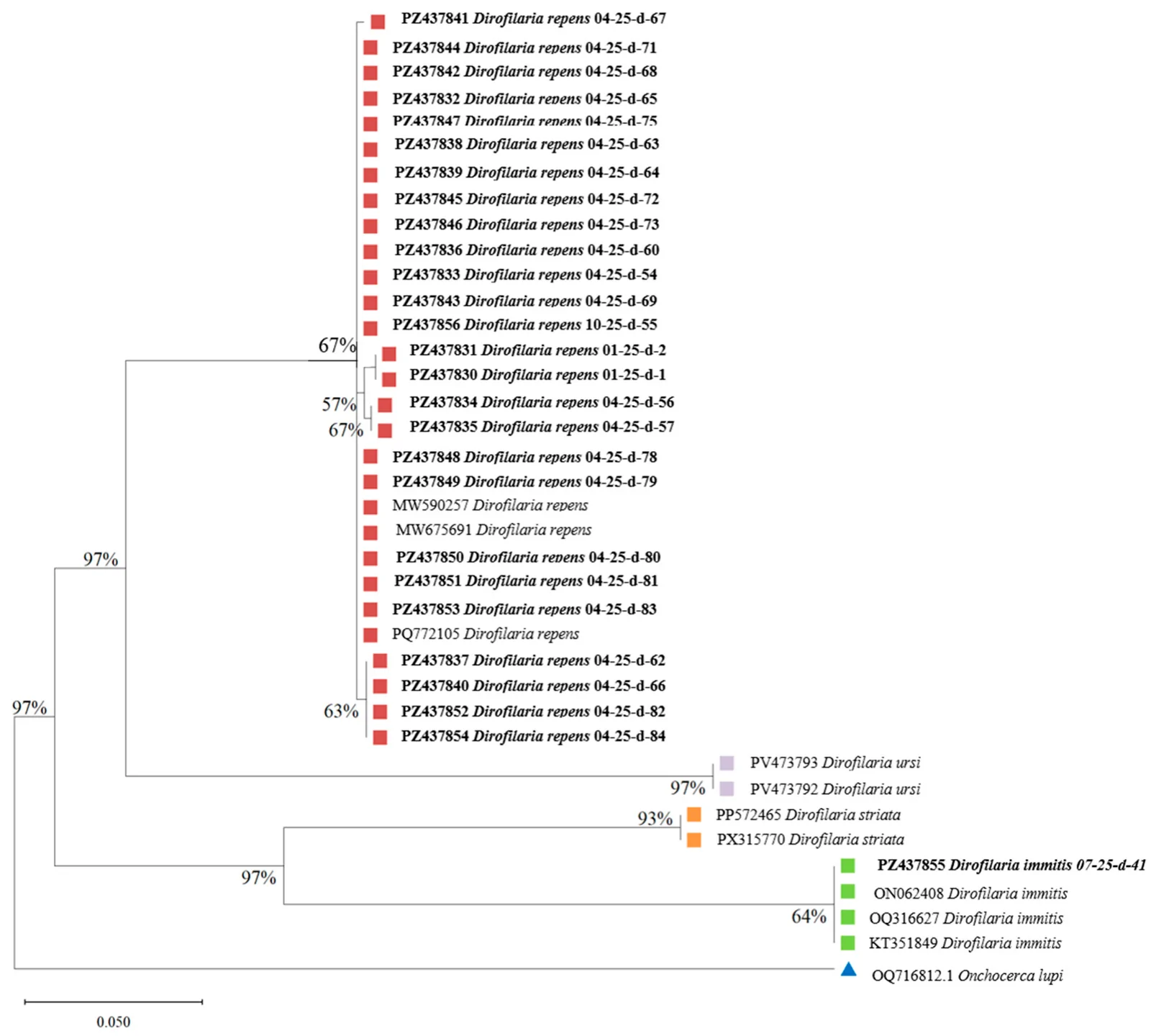
Maximum Likelihood phylogenetic tree of *Dirofilaria* spp. based on partial mitochondrial *cox1* gene sequences. The tree was constructed in MEGA11 using sequences aligned with CLUSTALW. Bootstrap values are shown at the nodes. *Onchocerca lupi* was used as an outgroup.

The Maximum Likelihood tree showed a clear separation of the analyzed sequences into species-specific clusters (Figure 4). The tree was rooted using *Onchocerca lupi* (OQ716812.1) as the outgroup, which formed a distinct external branch. All *D. repens* isolates obtained in this study clustered together with the reference sequences MW590257, MW675691, and PQ772105, confirming their species identification as *D. repens*.

Within the *D. repens* clade, the analyzed isolates demonstrated close genetic relationships, as indicated by the generally short internal branches. The entire *D. repens* clade formed a well-supported monophyletic group with a bootstrap value of 97%. Several weakly to moderately supported internal subdivisions were observed, with bootstrap values ranging from 57% to 67%. Among them, isolates PZ437837, PZ437840, PZ437852, and PZ437854 formed a distinct internal subgroup supported by a bootstrap value of 63%.

The isolate PZ437855 (*D. immitis* 07-25-d-41), obtained from Oral city, West Kazakhstan region, clustered with reference sequences of *D. immitis* (ON062408, OQ316627, and KT351849). This grouping confirmed the molecular identification of this isolate as *D. immitis*. The *D. immitis* cluster was supported by a bootstrap value of 64%.

Other *Dirofilaria* species included in the analysis formed separate clusters. The sequences of *D. ursi* clustered together with high bootstrap support (97%), forming the lineage most closely related to the *D. repens* clade. The broader grouping containing *D. striata* and *D. immitis* was supported by a bootstrap value of 97%, indicating a clear separation from the *D. repens–D. ursi* lineage. The separation of *D. repens*, *D. immitis, D. ursi*, and *D. striata* into distinct clades indicates that the analyzed *cox1* fragment provided sufficient phylogenetic resolution for species-level differentiation within the genus *Dirofilaria*.

### 3.4. Pairwise Evolutionary Divergence

Pairwise analysis of the mitochondrial *cox1* sequences revealed low intraspecific variation and substantially greater divergence among *Dirofilaria* spp. (Table S1). Within *D. repens*, pairwise evolutionary distances ranged from 0.00000 to 0.00672 substitutions per site, with a mean distance of approximately 0.00138. Most comparisons showed zero divergence, indicating that many isolates shared identical *cox1* sequences. The highest intraspecific distance was observed between isolate PZ437854 and the first two *D. repens* isolates, reaching 0.00672 substitutions per site. Several other isolates, including PZ437837, PZ437840, and PZ437852, also showed slightly elevated but still low divergence relative to the predominant sequence type.

No sequence divergence was detected between the two *D. striata* sequences, between the two *D. ursi* sequences, or among the four *D. immitis* sequences. In contrast, interspecific distances were considerably higher. Divergence between *D. repens* and *D. striata* ranged from 0.09232 to 0.12773, while divergence between *D. repens* and *D. ursi* ranged from 0.09393 to 0.12320. The distances between *D. repens* and *D. immitis* were higher, ranging from 0.12979 to 0.16927.

Among the remaining species, pairwise divergence ranged from 0.12225 to 0.13256 between *D. striata* and *D. immitis*, from 0.15964 to 0.16198 between *D. striata* and *D. ursi*, and from 0.16940 to 0.18554 between *D. ursi* and *D. immitis*. The greatest interspecific divergence was therefore observed between *D. ursi* and *D. immitis*.

## 4. Discussion

Dirofilariosis remains an important vector-borne zoonotic disease in many regions of the world [1,2,63,64,65,66,67,68,69,70,71,72]. However, data on the molecular prevalence of *Dirofilaria* spp. among dogs in Kazakhstan remain extremely limited. The country’s vast territory, diverse climatic zones, the presence of both owned and stray dog populations, and the potential distribution of competent mosquito vectors create favorable conditions for the circulation and transmission of *Dirofilaria* spp.

Although molecular epidemiological data on canine dirofilariosis in Kazakhstan remain scarce, the present study provides one of the first large-scale molecular assessments of *Dirofilaria* spp. circulation in dogs across multiple regions of the country, thereby contributing important baseline data for understanding the epidemiology of this zoonosis in Central Asia.

The predominance of *D. repens* observed in the present study is consistent with the epidemiological pattern reported from neighboring countries and indicates that this species currently represents the principal canine *Dirofilaria* species circulating in Kazakhstan. This species is of considerable public health importance, as it is recognized as the principal causative agent of human dirofilariosis in Europe and has been reported with increasing frequency across an expanding geographical range in recent years [8,73,74].

Given that dogs serve as the definitive hosts of *D. repens*, the detection of infected animals indicates the continued circulation of the parasite and the potential risk of transmission to humans in areas where competent mosquito vectors are present [9,43]. The predominance of *D. repens* has also been reported in several Eastern European countries, including Ukraine, where this species represents the majority of canine dirofilariosis cases [75].

Similar patterns have been observed in the Russian Federation. Large-scale molecular studies involving more than 10,000 dogs from different regions of Russia reported *D. repens* prevalence rates ranging from 7.52% to 7.69% among examined animals [35,36]. In regions bordering Kazakhstan, the prevalence varied from 0.0% to 11.5% [35]. The similarity between the epidemiological patterns observed in Kazakhstan and neighboring regions of Russia may reflect shared ecological conditions, common mosquito fauna, and unrestricted movement of reservoir hosts across national borders, suggesting the existence of transboundary transmission zones. In contrast, *D. immitis* appears to occur much less frequently in Russia. For example, in Vladivostok, this species was detected in only one dog among 782 examined animals (1/782) [76].

The higher diagnostic sensitivity of PCR compared with the modified Knott test observed in this study confirms the value of molecular methods for detecting low-level microfilaremia and occult infections that may remain undetected by conventional microscopy. These findings indicate the higher diagnostic sensitivity of molecular methods compared with conventional microscopy. The additional PCR-positive cases may be explained by the presence of dogs with low-level microfilaremia or occult infections, in which circulating microfilariae are absent or present in numbers below the detection limit of microscopic methods [77]. These findings support the incorporation of molecular diagnostic methods into routine epidemiological surveillance programmes, particularly in regions where the true prevalence of canine dirofilariosis is likely to be underestimated by microscopy alone.

The observed geographical heterogeneity suggests that local ecological and environmental factors play an important role in shaping transmission intensity across Kazakhstan. The markedly higher prevalence observed in Aktobe suggests regional differences in transmission, likely influenced by local environmental conditions and mosquito vector abundance.

The higher prevalence among shelter dogs probably reflects greater cumulative exposure to mosquito vectors together with limited implementation of preventive antiparasitic measures. The higher prevalence in dogs older than three years is biologically plausible because the long lifespan of adult *Dirofilaria* worms increases the probability of infection with cumulative exposure. No significant difference was detected between male and female dogs, indicating that sex does not appear to influence susceptibility to infection. The apparent increase in prevalence during winter should not be interpreted as evidence of increased winter transmission because infections are chronic and the observed pattern most likely reflects the temporal distribution of sampled animals.

Overall, the present findings demonstrate that *D. repens* is established in several regions of Kazakhstan, although transmission intensity appears to vary considerably across the country. This geographical heterogeneity is likely influenced by climatic conditions, mosquito abundance, local ecological characteristics, and differences in dog population structure.

The occurrence of infected dogs in multiple regions also has important veterinary and public health implications. Dogs represent the principal reservoir hosts for human infection; therefore, regular surveillance of both owned and shelter dogs should be considered an important component of national control programmes. Early detection and preventive treatment of infected dogs may reduce parasite circulation and consequently decrease the risk of human exposure.

The phylogenetic analysis based on the mitochondrial *cox1* gene confirmed the species identity of the *Dirofilaria* isolates obtained from dog blood samples in Kazakhstan. The isolates generated in the present study clustered with reference sequences of the corresponding *Dirofilaria* species from GenBank, demonstrating that the analyzed *cox1* fragment provides sufficient phylogenetic resolution for reliable species-level identification of *D. repens* and *D. immitis* [78,79,80]. The mitochondrial *cox1* gene was selected because it is the most widely used marker for the identification and phylogenetic analysis of *Dirofilaria* spp., allowing direct comparison with numerous reference sequences available in GenBank. Future studies using additional markers (e.g., 12S rRNA, *ITS* regions, complete mitochondrial genomes, or multilocus approaches) may provide greater resolution of the genetic diversity and phylogeography of *Dirofilaria* populations in Kazakhstan.

The predominance of *D. repens* among the sequenced isolates is consistent with the epidemiological findings of the present study and further supports that this species is currently the dominant canine *Dirofilaria* spp. circulating in Kazakhstan. In the phylogenetic tree, the Kazakhstani *D. repens* isolates grouped together with reference *D. repens* sequences, forming a clearly separated clade. This clustering confirms the molecular identification of the isolates and indicates that the Kazakhstani sequences belong to the globally recognized genetic lineage of *D. repens*. The clustering pattern also suggests a close genetic relationship between the Kazakhstani isolates and previously reported *D. repens* sequences from other geographic areas.

Only limited genetic differentiation was observed among the Kazakhstani isolates based on the analyzed *cox1* fragment. However, the moderate bootstrap support values observed within the *D. repens* clade indicate limited genetic variability among the analyzed isolates based on the examined *cox1* fragment. This pattern may indicate the circulation of closely related haplotypes over relatively large geographic distances. Alternatively, the limited variability may reflect the conservative nature of the analyzed mitochondrial fragment rather than the true genetic diversity of the parasite population. Therefore, analyses based on additional mitochondrial and nuclear markers will be necessary to characterize the population structure and evolutionary history of *Dirofilaria* spp. in Kazakhstan more comprehensively.

The isolate PZ437855, obtained from Oral City, West Kazakhstan Region, clustered with reference sequences of *D. immitis*. This result confirms the presence of *D. immitis* in the examined dog population of western Kazakhstan. The clear separation between the *D. repens* and *D. immitis* clades further demonstrates the suitability of the cox1 marker for reliable discrimination between these two medically and veterinary important species [81].

The inclusion of additional *Dirofilaria* spp. and an appropriate outgroup provided robust phylogenetic support for the placement of the Kazakhstani isolates and confirmed their clear genetic separation from other members of the genus. The prevalence observed in Kazakhstan is generally consistent with reports from neighboring Central Asian countries, although considerable regional variation exists. Higher prevalence has been reported in Uzbekistan, whereas substantially lower infection rates have been documented in Kyrgyzstan [39,40,41].

The pairwise-distance analysis further confirmed the molecular identification of the analysed isolates. The low intraspecific divergence observed among *D. repens* isolates indicates limited mitochondrial variability within the analysed *cox1* fragment and suggests the circulation of closely related haplotypes in Kazakhstan. In contrast, the substantially greater interspecific divergence demonstrates that the *cox1* marker provides reliable discrimination among *D. repens*, *D. immitis*, *D. striata*, and *D. ursi*, supporting the species-level clustering observed in the phylogenetic analysis.

These differences are likely attributable to climatic conditions, mosquito abundance, dog population structure, sampling strategies, and diagnostic approaches used across individual studies. Nevertheless, the available evidence indicates that *Dirofilaria* spp. is widely distributed throughout Central Asia and continues to circulate in canine populations across the region. The lack of molecular epidemiological data from Tajikistan and Turkmenistan further highlights the need for coordinated regional surveillance of canine dirofilariosis in Central Asia.

In contrast to Kazakhstan and several neighboring countries, where *D. repens* predominates, *D. immitis* remains the dominant species in many parts of Asia, including China, Pakistan, Turkey, and Iran [30,62,82,83]. These contrasting epidemiological patterns most likely reflect regional differences in climate, mosquito fauna, host populations, and ecological conditions affecting parasite transmission.

The distribution of *Dirofilaria* spp. is determined not only by the presence of suitable definitive hosts but also by climatic conditions that enable the development of infective larvae within mosquito vectors [84]. It is noteworthy that the Aktobe Region was among the areas most severely affected by the large-scale floods that occurred in Kazakhstan in 2024 [85]. The formation of numerous temporary water bodies and the prolonged persistence of high humidity may have created favorable conditions for an increase in mosquito populations, thereby facilitating more intensive transmission of *Dirofilaria* spp. among dogs in the region. Although the direct impact of flooding on infection prevalence requires further investigation, the present findings suggest a possible association between hydrometeorological changes and the emergence of a local focus of canine dirofilariosis.

The successful transmission of *Dirofilaria* spp. depends on the presence of competent mosquito vectors. Several mosquito genera known to transmit canine dirofilariosis, including *Aedes*, *Culex*, and *Anopheles*, are widely distributed in Kazakhstan and may contribute to the local transmission of the parasite [86,87,88]. Although mosquito surveillance was beyond the scope of the present study, entomological monitoring has recently been initiated by our research group to investigate the diversity of mosquito species, their geographical distribution, and their potential role in the transmission of *Dirofilaria* spp. in Kazakhstan. The results of these investigations will provide important information on vector ecology and the epidemiology of canine dirofilariosis in the country.

The widespread occurrence of competent mosquito vectors, together with favorable climatic conditions during the warm season, suggests that local transmission of canine dirofilariosis is biologically plausible in several regions of Kazakhstan. Although the introduction of infected dogs from other regions cannot be completely excluded, the detection of *Dirofilaria* spp. in wolves reported previously [44,45] and our preliminary unpublished observations in red foxes support the existence of natural transmission cycles involving wildlife reservoirs.

The findings of the present study also have important veterinary and One Health implications. Because dogs represent the principal reservoir hosts for human dirofilariosis, continuous surveillance of canine populations may serve as an effective early warning system for emerging zoonotic transmission. Close collaboration among veterinarians, physicians, parasitologists, and entomologists will be essential for monitoring the spread of the parasite and developing evidence-based prevention and control strategies in Kazakhstan.

Several limitations of the present study should be acknowledged. First, sampling was restricted to selected regions of Kazakhstan and therefore may not fully represent the nationwide distribution of *Dirofilaria* spp. Sampling was restricted to major urban centers and the study population may not be fully representative of the entire canine population of Kazakhstan, particularly dogs living in rural areas. The possibility that some infected dogs had been introduced from other regions cannot be excluded. Similarly, the role of wildlife reservoirs requires further investigation. Second, mosquito vectors were not investigated, preventing a comprehensive assessment of transmission intensity and vector involvement in the studied areas. Consequently, the mosquito species involved in local transmission and their seasonal dynamics remain unknown. Finally, seasonal patterns of infection were not evaluated using a balanced longitudinal sampling design. Therefore, the higher prevalence observed during winter should be interpreted with caution, as it may partly reflect the temporal and regional distribution of the sampled animals, including the concentration of positive samples from the Aktobe region, rather than true seasonal variation. Future studies should integrate molecular surveillance of dogs with entomological monitoring of mosquito populations and investigations of wildlife reservoirs using a One Health approach to better understand the transmission dynamics of canine dirofilariosis in Kazakhstan.

## 5. Conclusions

This study provides the first molecular evidence of the circulation of *D. repens* and *D. immitis* in dogs from Kazakhstan and represents the most comprehensive molecular survey of canine dirofilariosis conducted in the country to date. An overall prevalence of 3.6% was detected, with *D. repens* identified as the predominant species and the highest infection rate observed in the Aktobe Region. Phylogenetic analysis confirmed species identity and revealed a high genetic similarity between Kazakhstani isolates and those reported from other parts of Europe and Asia. These findings demonstrate the ongoing circulation of *Dirofilaria* spp. in canine populations, highlight the value of molecular diagnostics for epidemiological surveillance, and emphasize the need for continued monitoring of this zoonotic infection in Kazakhstan and across Central Asia. Future research should adopt a “*One Health*” approach and incorporate studies of wild canids (e.g., foxes and wolves), mosquito vectors, and human populations to improve our understanding of the epidemiology, transmission dynamics, and zoonotic risks associated with *Dirofilaria* spp. in the region.

## Figures and Tables

**Table 1:** Prevalence of *Dirofilaria* spp. according to the investigated factors.

Variable	No. Examined	Positive Samples, *n*	Prevalence, % (95% CI)	Statistic	Exact *p*-Value	*p*-Value
City						
Astana	75	2	2.7 (0.3–9.3)	-	8.33 × 10^−15^	*p* < 0.0001
Almaty	50	0				
Aktobe	103	23	22.3 (14.7–31.6)			
Oral	55	1	1.8 (0.05–9.7)			
Shymkent	104	0				
Turkestan	140	0				
Taraz	100	0				
Kostanay	60	1	1.7 (0.04–9.0)			
Karaganda	14	0				
Pavlodar	50	0				
Season						
Spring	169	1	0.6 (0.01–3.2)	-	1.84 × 10^−7^	*p* < 0.0001
Summer	155	1	0.6 (0.02–3.5)			
Autumn	130	0				
Winter	297	25	8.4 (5.5–12.2)			
Sex						
Male	396	15	3.8 (2.1–6.2)	χ^2^ = 0.00; df = 1	1.000	*p* = 1.000
Female	325	12	3.7 (2.0–6.3)			
N/A	30	0				
Living condition						
Companion	545	4	0.7 (0.2–1.8)	χ^2^ = 43.97; df = 1	3.33 × 10^−11^	*p* < 0.0001
Shelter	206	23	11.2 (7.2–16.3)			
Age class						
6–12 month	87	0		-	2.87 × 10^−5^	*p* < 0.0001
12 month to 3 years	258	3	1.2% (0.2–3.4)			
>3 years	304	24	7.9 (5.2–11.5)			
N/A	102	0				
Total	751	27	3.6 (2.4–5.2)			

df—degrees of freedom.

**Table 2:** Comparison of *Dirofilaria* spp. detection by the modified Knott test and PCR in dogs from different regions of Kazakhstan.

City	Modified Knott’s Test	PCR		
	Positive, *n* (%)	Positive, *n* (%)	*D. immitis*	*D. repens*
Astana	1(1.3)	2 (2.7)	0	2
Almaty	0	0	0	0
Aktobe	12(11.65)	23 (22.3)	0	23
Oral	1(1.8)	1 (1.8)	1	0
Shymkent	0	0	0	0
Turkestan	0	0	0	0
Taraz	0	0	0	0
Kostanay	1(1.7)	1 (1.7)	0	1
Karaganda	0	0	0	0
Pavlodar	0	0	0	0
Total	15 (2.0)	27 (3.6)	1	26

## Data Availability

The original contributions presented in this study are included in the article/Supplementary Material. Further inquiries can be directed to the corresponding author.

## Supplementary Materials

The following supporting information can be downloaded at: https://www.mdpi.com/article/10.3390/ani16162612/s1, Table S1: Intraspecific and interspecific pairwise evolutionary divergence among *Dirofilaria* spp. based on mitochondrial cox1 sequences.

## Abbreviations

The following abbreviations are used in this manuscript:

bp	base pair
CI	confidence interval
cox1	cytochrome c oxidase subunit 1
DNA	deoxyribonucleic acid
OR	odds ratio
TBE	Tris-borate-EDTA buffer
UV	ultraviolet
