# Supplementary material for: Geographical Distribution and Molecular Characterization of Dirofilaria spp. in Dogs Across Kazakhstan
Source: Animals (Basel). 2026 Aug 20;16(16):2612. doi: 10.3390/ani16162612 (PMC13509504; doi:10.3390/ani16162612)
Supplement: Supplementary file 1 [file animals-16-02612-s001.zip › animals-4435635-supplementary.pdf]

Table S1. Intraspecific and interspecific pairwise evolutionary divergence among *Dirofilaria* spp. based on mitochondrial *cox1* sequences.

1	PZ437831 *D.repens* 01-25-d-2																																					
2	PZ437830 *D.repens* 01-25-d-1	0,00000																																				
3	MW590257 *D.repens*	0,00260	0,00260																																			
4	PZ437833 *D.repens* 04-25-d-54	0,00260	0,00260	0,00000																																		
5	MW675691 *D.repens*	0,00260	0,00260	0,00000	0,00000																																	
6	PQ772105 *D.repens*	0,00260	0,00260	0,00000	0,00000	0,00000																																
7	PZ437850_Dirofilaria_repens_04-25-d-80	0,00260	0,00260	0,00000	0,00000	0,00000	0,00000																															
8	PZ437848 *D.repens* 04-25-d-78	0,00260	0,00260	0,00000	0,00000	0,00000	0,00000	0,00000																														
9	PZ437839_Dirofilaria_repens_04-25-d-64	0,00296	0,00296	0,00000	0,00000	0,00000	0,00000	0,00000	0,00000																													
10	PZ437836 *D.repens* 04-25-d-60	0,00303	0,00303	0,00000	0,00000	0,00000	0,00000	0,00000	0,00000	0,00000																												
11	PZ437846 *D.repens* 04-25-d-73	0,00260	0,00260	0,00000	0,00000	0,00000	0,00000	0,00000	0,00000	0,00000	0,00000																											
12	PZ437853 *D.repens* 04-25-d-83	0,00264	0,00264	0,00000	0,00000	0,00000	0,00000	0,00000	0,00000	0,00000	0,00000	0,00000																										
13	PZ437849 *D.repens* 04-25-d-79	0,00291	0,00291	0,00000	0,00000	0,00000	0,00000	0,00000	0,00000	0,00000	0,00000	0,00000	0,00000																									
14	PZ437838 *D.repens* 04-25-d-63	0,00310	0,00310	0,00000	0,00000	0,00000	0,00000	0,00000	0,00000	0,00000	0,00000	0,00000	0,00000	0,00000																								
15	PZ437832 *D.repens* 04-25-d-65	0,00310	0,00310	0,00000	0,00000	0,00000	0,00000	0,00000	0,00000	0,00000	0,00000	0,00000	0,00000	0,00000	0,00000																							
16	PZ437856 *D.repens* 10-25-d-55	0,00319	0,00319	0,00000	0,00000	0,00000	0,00000	0,00000	0,00000	0,00000	0,00000	0,00000	0,00000	0,00000	0,00000	0,00000																						
17	PZ437842 *D.repens* 04-25-d-68	0,00294	0,00294	0,00000	0,00000	0,00000	0,00000	0,00000	0,00000	0,00000	0,00000	0,00000	0,00000	0,00000	0,00000	0,00000	0,00000																					
18	PZ437851 *D.repens* 04-25-d-81	0,00295	0,00295	0,00000	0,00000	0,00000	0,00000	0,00000	0,00000	0,00000	0,00000	0,00000	0,00000	0,00000	0,00000	0,00000	0,00000	0,00000																				
19	PZ437845 *D.repens* 04-25-d-72	0,00298	0,00298	0,00000	0,00000	0,00000	0,00000	0,00000	0,00000	0,00000	0,00000	0,00000	0,00000	0,00000	0,00000	0,00000	0,00000	0,00000	0,00000																			
20	PZ437844 *D.repens* 04-25-d-71	0,00300	0,00300	0,00000	0,00000	0,00000	0,00000	0,00000	0,00000	0,00000	0,00000	0,00000	0,00000	0,00000	0,00000	0,00000	0,00000	0,00000	0,00000	0,00000																		
21	PZ437847 *D.repens* 04-25-d-75	0,00302	0,00302	0,00000	0,00000	0,00000	0,00000	0,00000	0,00000	0,00000	0,00000	0,00000	0,00000	0,00000	0,00000	0,00000	0,00000	0,00000	0,00000	0,00000	0,00000																	
22	PZ437843 *D.repens* 04-25-d-69	0,00342	0,00342	0,00000	0,00000	0,00000	0,00000	0,00000	0,00000	0,00000	0,00000	0,00000	0,00000	0,00000	0,00000	0,00000	0,00000	0,00000	0,00000	0,00000	0,00000	0,00000																
23	PZ437835 *D.repens* 04-25-d-57	0,00300	0,00300	0,00365	0,00296	0,00365	0,00365	0,00230	0,00190	0,00189	0,00367	0,00201	0,00224	0,00216	0,00217	0,00217	0,00238	0,00209	0,00218	0,00365	0,00203	0,00221	0,00238															
24	PZ437834 *D.repens* 04-25-d-56	0,00260	0,00260	0,00198	0,00256	0,00198	0,00198	0,00205	0,00198	0,00220	0,00224	0,00198	0,00203	0,00217	0,00227	0,00227	0,00238	0,00219	0,00219	0,00221	0,00222	0,00223	0,00244	0,00000														

25	PZ437841 *D.repens* 04-25-d-67	0,00527	0,00527	0,00182	0,00258	0,00182	0,00182	0,00206	0,00182	0,00000	0,00000	0,00183	0,00204	0,00000	0,00000	0,00000	0,00000	0,00000	0,00000	0,00000	0,00000	0,00000	0,00000	0,00200	0,00400													
26	PZ437837 *D.repens* 04-25-d-62	0,00597	0,00597	0,00226	0,00293	0,00226	0,00226	0,00229	0,00226	0,00226	0,00229	0,00226	0,00226	0,00226	0,00233	0,00233	0,00238	0,00226	0,00226	0,00226	0,00228	0,00229	0,00251	0,00457	0,00453	0,00226												
27	PZ437840 *D.repens* 04-25-d-66	0,00606	0,00606	0,00240	0,00298	0,00240	0,00240	0,00240	0,00240	0,00240	0,00241	0,00240	0,00240	0,00240	0,00245	0,00245	0,00251	0,00240	0,00240	0,00240	0,00240	0,00240	0,00265	0,00482	0,00482	0,00240	0,00000											
28	PZ437852 *D.repens* 04-25-d-82	0,00601	0,00601	0,00238	0,00295	0,00238	0,00238	0,00238	0,00238	0,00238	0,00241	0,00238	0,00238	0,00238	0,00245	0,00245	0,00251	0,00238	0,00238	0,00238	0,00239	0,00240	0,00265	0,00480	0,00478	0,00238	0,00000	0,00000										
29	PZ437854 *D.repens* 04-25-d-84	0,00672	0,00672	0,00307	0,00329	0,00307	0,00307	0,00307	0,00307	0,00307	0,00307	0,00307	0,00307	0,00307	0,00307	0,00307	0,00307	0,00307	0,00307	0,00307	0,00307	0,00307	0,00314	0,00618	0,00618	0,00307	0,00000	0,00000	0,00000									
30	PP572465 *D.striata*	0,11634	0,11634	0,09965	0,10938	0,09965	0,09965	0,10608	0,09232	0,10334	0,10036	0,09543	0,10793	0,11451	0,10580	0,10580	0,11637	0,11396	0,11739	0,10180	0,10402	0,11282	0,11117	0,10232	0,10760	0,10208	0,12446	0,12310	0,12179	0,12773								
31	PX315770 *D.striata*	0,11634	0,11634	0,09767	0,10938	0,09767	0,09767	0,10608	0,09232	0,10334	0,09799	0,09543	0,10793	0,11451	0,10580	0,10580	0,11637	0,11396	0,11739	0,09939	0,10402	0,11282	0,11117	0,10131	0,10760	0,10208	0,12446	0,12310	0,12179	0,12773	0,00000							
32	PV473793 *D.ursi*	0,12207	0,12207	0,11158	0,11535	0,11158	0,11158	0,11902	0,10590	0,09857	0,09393	0,11327	0,11329	0,11625	0,10497	0,10497	0,10260	0,11158	0,11455	0,09844	0,10262	0,10628	0,10522	0,10026	0,12320	0,11639	0,10676	0,10273	0,10621	0,10628	0,15964	0,16198						
33	PV473792 *D.ursi*	0,12207	0,12207	0,11158	0,11535	0,11158	0,11158	0,11902	0,10590	0,09857	0,09393	0,11327	0,11329	0,11625	0,10497	0,10497	0,10260	0,11158	0,11455	0,09844	0,10262	0,10628	0,10522	0,10026	0,12320	0,11639	0,10676	0,10273	0,10621	0,10628	0,15964	0,16198	0,00000					
34	KT351849 *D.immitis*	0,14486	0,14486	0,12979	0,15437	0,12979	0,12979	0,14519	0,13357	0,13565	0,13242	0,13199	0,14407	0,14397	0,14011	0,14011	0,15650	0,14371	0,14777	0,13352	0,13931	0,14338	0,13658	0,14076	0,14146	0,13529	0,15267	0,15174	0,15466	0,16475	0,12831	0,12857	0,1694	0,16945				
35	ON062408 *D.immitis*	0,14992	0,14992	0,13434	0,15832	0,13434	0,13434	0,14777	0,13534	0,13565	0,13640	0,13384	0,14407	0,14397	0,14011	0,14011	0,15650	0,14371	0,14777	0,13752	0,13931	0,14338	0,13658	0,14283	0,14392	0,13751	0,15267	0,15174	0,15466	0,16475	0,13256	0,13224	0,18554	0,18554	0,00000			
36	OQ316627 *D.immitis*	0,14992	0,14992	0,13177	0,15832	0,13177	0,13177	0,14777	0,13568	0,13565	0,13242	0,13420	0,14407	0,14397	0,14011	0,14011	0,15650	0,14371	0,14777	0,13352	0,13931	0,14338	0,13658	0,14076	0,14392	0,13751	0,15267	0,15174	0,15466	0,16475	0,13038	0,13058	0,17011	0,17011	0,00000	0,00000		
37	PZ437855 *D.immitis* 07-25-d-41	0,15738	0,15738	0,15083	0,16927	0,15083	0,15083	0,15475	0,15083	0,15083	0,15083	0,15083	0,15083	0,15083	0,15083	0,15083	0,15650	0,15083	0,15083	0,15083	0,15083	0,15083	0,14215	0,15083	0,15083	0,15083	0,15593	0,15700	0,15700	0,16475	0,12225	0,12225	0,17197	0,17197	0,00000	0,00000	0,00000	
